# Research priorities for neuroimmunology: identifying the key research questions to be addressed by 2030

**DOI:** 10.12688/wellcomeopenres.16997.1

**Published:** 2021-07-29

**Authors:** Georgina MacKenzie, Sumithra Subramaniam, Lindsey J Caldwell, Denise Fitzgerald, Neil A Harrison, Soyon Hong, Sarosh R Irani, Golam M Khandaker, Adrian Liston, Veronique E Miron, Valeria Mondelli, B Paul Morgan, Carmine Pariante, Divya K Shah, Leonie S Taams, Jessica L Teeling, Rachel Upthegrove

**Affiliations:** 1 Wellcome Trust, London, NW1 2BE, UK; 2 UK Dementia Research Institute Headquarters, London, UK; 3 Wellcome-Wolfson Institute for Experimental Medicine, Queen's University Belfast, Belfast, UK; 4 Cardiff University Brain Research Imaging Centre (CUBRIC), Cardiff University, Cardiff, UK; 5 Division of Psychological Medicine and Clinical Neuroscience, Cardiff University, Cardiff, UK; 6 UK Dementia Research Institute, Institute of Neurology, University College London, London, UK; 7 Oxford Autoimmune Neurology Group, Nuffield Department of Clinical Neurosciences, University of Oxford, Oxford, UK; 8 Department of Neurology, Oxford University Hospitals NHS Foundation Trust, Oxford, UK; 9 MRC integrative Epidemiology Unit, Population Health Sciences, Bristol Medical School, University of Bristol, Bristol, UK; 10 Centre for Academic Mental Health, Population Health Sciences, Bristol Medical School, University of Bristol, Bristol, UK; 11 Department of Psychiatry, University of Cambridge School of Clinical Medicine, University of Cambridge, Cambridge, UK; 12 Laboratory of Lymphocyte Signalling and Development, The Babraham Institute, Cambridge, UK; 13 UK Dementia Research Institute at The University of Edinburgh, Centre for Discovery Brain Sciences, University of Edinburgh, Edinburgh, UK; 14 National Institute for Health Research, Mental Health Biomedical Research Centre, South London and Maudsley NHS Foundation Trust and King's College London, London, UK; 15 Department of Psychological Medicine, Institute of Psychiatry, Psychology and Neuroscience, King's College London, London, UK; 16 UK Dementia Research Institute, Cardiff University, Cardiff, UK; 17 Systems Immunity Research Institute, Cardiff University, Cardiff, UK; 18 Centre for Inflammation Biology & Cancer Immunology, Department of Inflammation Biology, School of Immunology & Microbial Sciences, King's College London, London, UK; 19 Biological Sciences, Faculty of Natural and Environmental Sciences, University of Southampton, Southampton, UK; 20 Early Intervention Service, Birmingham Womens and Children’s NHS Foundation Trust, Birmingham, UK; 21 Institute for Mental Health, University of Birmingham, Birmingham, UK

**Keywords:** Peripheral nervous system, central nervous system, psychoneuroimmunology, immunopsychiatry, behaviour, mental health, inflammation, neuroimmune interactions

## Abstract

Neuroimmunology in the broadest sense is the study of interactions between the nervous and the immune systems. These interactions play important roles in health from supporting neural development, homeostasis and plasticity to modifying behaviour. Neuroimmunology is increasingly recognised as a field with the potential to deliver a significant positive impact on human health and treatment for neurological and psychiatric disorders. Yet, translation to the clinic is hindered by fundamental knowledge gaps on the underlying mechanisms of action or the optimal timing of an intervention, and a lack of appropriate tools to visualise and modulate both systems. Here we propose ten key disease-agnostic research questions that, if addressed, could lead to significant progress within neuroimmunology in the short to medium term. We also discuss four cross-cutting themes to be considered when addressing each question: i) bi-directionality of neuroimmune interactions; ii) the biological context in which the questions are addressed (e.g. health vs disease vs across the lifespan); iii) tools and technologies required to fully answer the questions; and iv) translation into the clinic. We acknowledge that these ten questions cannot represent the full breadth of gaps in our understanding; rather they focus on areas which, if addressed, may have the most broad and immediate impacts. By defining these neuroimmunology priorities, we hope to unite existing and future research teams, who can make meaningful progress through a collaborative and cross-disciplinary effort.

## Disclaimer

The views expressed in this article are those of the author(s). Publication in Wellcome Open Research does not imply endorsement by Wellcome.

## Introduction

Investigation of interactions between the nervous and immune systems, referred to here as neuroimmunology, is a dynamic, interdisciplinary field involving (but not limited to) immunology, neuroscience, inflammation biology, neurology, psychiatry and psychology. Neuroimmunology represents a rapidly expanding area of research with a high potential to improve human health, catalysed by increasing evidence implicating the immune system in neurological and psychiatric development and disorders and, conversely, the role of the nervous system in modifying immune function. These interactions are dynamic and diverse, ranging from health and homeostasis, across the lifespan, from development to old age and during disease. Closing the gap in our knowledge will increase our fundamental understanding of communication between two complex systems and how neuroimmune interactions change over time, how they influence our behaviour and what happens in neurological and psychiatric disorders.

Progress towards realising the translational potential of neuroimmunology has, at times, been hindered by gaps in basic mechanistic understanding and limited tools to monitor, measure and modulate cells and molecules of the central nervous system (CNS). Better understanding of the multiple interactions between cell types of the nervous and immune systems is required, both within the CNS and the periphery. Furthermore, the role of cells at the interface of the two systems, such as border-associated macrophages and immune cells that reside or circulate within and beyond the CNS, is becoming increasingly appreciated. This highlights the importance of understanding dynamic neuroimmune interactions in the broadest sense. It is important to consider when and where these interactions are biologically advantageous or dysfunctional. For example, microglia, the tissue-resident macrophages and most abundant immunological cell type in the CNS, play a vital role in supporting healthy nervous system development and homeostasis; however, they can also contribute to neuroinflammation and neuronal dysfunction/degeneration, depending on the context ^1^. Conversely, modulating neuronal activity or sensory experiences can modify microglial functional states ^2, 3^, providing an example of how multiple interactions between cell types in the CNS are critical for health and homeostasis.

While it has long been known that microbial infections can affect the nervous system through interactions with the immune system ^4^, the coronavirus disease 2019 (COVID-19) pandemic has highlighted how environmental influences can have an impact on neuroimmune interactions. In particular, it has been shown that a psychiatric diagnosis could be an independent risk factor for COVID-19, but equally that survivors of COVID-19 are at increased risk of a subsequent psychiatric or neurological diagnosis ^5^. As we begin to understand the neurological and psychiatric symptoms of long COVID, there has been increased interest in neuroimmune interactions and immune-to-brain communication, and how their homeostatic balance is affected by infection ^4, 6, 7^.

### Identifying the research priorities in neuroimmunology

Setting research priorities requires bringing together the relevant stakeholders to collectively determine which ‘uncertainties’ in a field are the most pressing issues to resolve through research. Identifying the key scientific questions can help a community galvanise around a common set of goals and facilitate the formation of new collaborations.

To accomplish this, and building off of earlier discussions ^8^, in October 2020, Wellcome brought together some of the leading neuroimmunology researchers in the UK, from across a range of institutions, career stages and clinical and scientific backgrounds. Members of the steering committee can be found in Table 1. The goal was to identify questions that, if addressed, would deliver a step change in our understanding in the short and medium term (five to 10 years) and, in turn, have a positive impact on human health. It was important that these questions were generated by the research community, with the greatest potential to maximise basic understanding and therapeutic advances, without being influenced by other factors or strategies. The aim was to focus on the key questions that would have the broadest and most immediate impact, and that would be comprehensive and inclusive for the whole neuroimmunology community while still providing meaningful and tangible scientific direction.

**Table 1.** Members of the steering committee involved in identifying and defining the research priorities in neuroimmunology.

Steering group member	Affiliation	Biography
Denise Fitzgerald	Professor of Neuroimmunology, Queen’s University Belfast	Professor Fitzgerald’s research examines how the immune system influences tissue damage and regeneration in the central nervous system with a focus on demyelinating diseases.
Neil Harrison	Clinical Professor of Neuroimaging, Cardiff University	Professor Harrison’s research investigates how the body's immune system interacts with the brain to alter human mood, motivation and cognition and contributes to common mental illnesses, particularly depression. He is the current President of the Psychoneurommunology Research Society (PNIRS).
Soyon Hong	Group leader, UK Dementia Research at University College London	Dr Hong studies how immune pathways and neuroglia interactions contribute to regional vulnerability of synaptic dysfunction and loss in Alzheimer’s and Parkinson’s diseases.
Sarosh R Irani	Head, Oxford Autoimmune Neurology Group and MRC Senior Clinical Fellow	Professor Irani’s work aims to understand distinctive clinical features and the underlying immunobiology in patients with autoantibody-mediated diseases of the nervous system.
Golam Khandaker	Professor of Psychiatry and Head of Immunopsychiatry and Experimental Medicine Programme, MRC Integrative Epidemiology Unit, University of Bristol	Professor Khandaker’s research focuses on identifying and validating novel immunological mechanisms and potential treatment targets for major psychiatric disorders, particularly depression and schizophrenia, using population-based data, genetic analysis, and early-phase clinical trials.
Adrian Liston	Senior Group Leader at Babraham Institute & VIB Center for Brain and Disease Research	Professor Liston’s research focuses on the role that the immune system plays in the tissues, with a particular emphasis on the brain in health, ageing and following traumatic brain injury.
Veronique Miron	MRC Senior Research Fellow and Group leader, UK Dementia Research at The University of Edinburgh	Dr Miron’s research focuses on the role of the innate immune system in brain health across the lifespan, in order to identify novel therapeutic strategies for patients suffering from neurodegenerative disorders.
Valeria Mondelli	Clinical Reader in Psychoneuroimmunology at King’s College London	Dr Mondelli’s research focuses on the interface between physical and mental health and aims to advance our understanding of the interaction between the immune and central nervous systems and to apply this knowledge to improve prevention and treatment strategies of patients suffering with psychiatric disorders.
B. Paul Morgan	Professor of Immunology, Systems Immunity URI and Dementia Research Institute, Cardiff	Professor Morgan’s work focusses on the roles of inflammation as a driver of neurodegeneration in dementias. His particular expertise is in the complement system.
Carmine Pariante	Professor of Biological Psychiatry at King’s College London	Professor Pariante has been studying the role of the immune system in psychiatric disorders for the last 30 years, with research in humans, animals and cellular models. He is the Editor in Chief of *Brain, Behavior and Immunity*, a scientific journal dedicated to both clinical and basic research in neuroimmunology and psychoneuroimmunology.
Leonie Taams	Professor of Immune Regulation & Inflammation at King’s College London	Professor Taams’s research is focused on the cellular and molecular mechanisms that initiate, perpetuate and regulate inflammation in humans, with a particular interest in inflammatory arthritis. She is also the Director of the Wellcome PhD Programme in “Neuro-Immune Interactions in Health and Disease” at King’s.
Jessica Teeling	Professor of Experimental Neuroimmunology at University of Southampton	Professor Teeling’s research focusses on the interactions between systemic inflammation and the central nervous system, and in particular inflammation caused by acute or chronic bacterial infections.
Rachel Upthegrove	Professor of Psychiatry and Youth Mental Health	Professor Upthegrove’s research focusses on the identification of underlying immune mechanisms and novel treatments for developing mental health disorders, with particular focus on early stages of schizophrenia. She sits on the Wellcome Trust Policy Advisory Committee, MRC Neurosciences and Mental Health Board and British Association for Psychopharmacology council.

To this end, the scope for this exercise covered fundamental biology through to clinical application, in both health (for instance, homeostasis, development, healthy aging and behaviour) and disease (such as dementia, multiple sclerosis, depression and schizophrenia). Broad definitions of the nervous and immune systems were maintained. The nervous system would include: neuronal synapses and surrounding glia, specific brain, spinal cord or retinal compartments, the peripheral nervous system and the cerebrospinal fluid. The immune system would include both the peripheral and CNS-resident innate and adaptive immune systems, and those at the interface with the nervous system, including cells associated with the blood brain barrier. In addition, it was considered important to be inclusive in language and terminology, whilst being able to communicate across diverse fields. Throughout, we refer to neuroimmunology as encompassing the full range of the two systems, from molecular and cellular interactions, through to immunopsychiatry and psychoneuroimmunology, thus inclusive of mind, brain and environment interactions.

A conscious decision was made to be disease-agnostic when setting the research questions, while recognising the importance of the clinical context, as discussed in detail later. As such, persons with lived experience were not included at this stage of the process, although we recognise their future participation is essential when setting priorities for specific conditions.

### Research priorities in neuroimmunology

**Figure 1.**
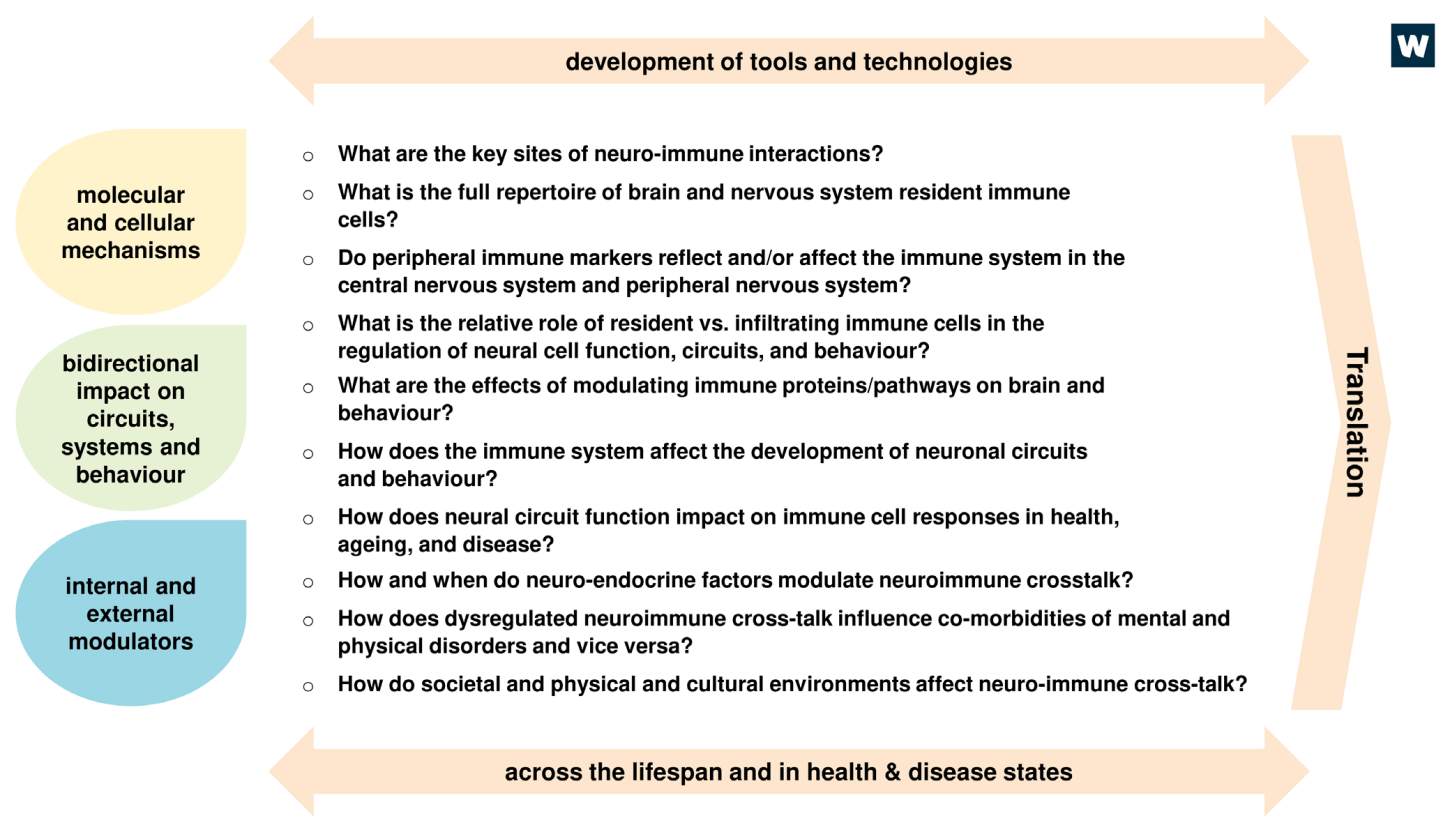
Research priorities in neuroimmunology. Ten key research questions were identified which, if addressed, would drive the field forward in the short to medium term and translate to a positive impact on human health. The ten questions can broadly be grouped into three categories reflecting the scale of analysis (e.g. molecules, circuits) and internal and external factors that influence, or are influenced by, neuroimmune interactions. Each of these questions will need to be addressed in the relevant contexts including across the lifespan and in health and disease, and may require the development or adoption of new tools and technologies to be successful. Addressing these questions will strengthen the fundamental knowledge base and ultimately drive translation (e.g. through identification of new targets, biomarkers).

By engaging with a broad spectrum of stakeholders in an open and transparent process, the following research priorities were identified collectively ( Figure 1).

Aiming to keep the research priorities broad and applicable to the diverse researchers in the field, four cross-cutting themes were discussed that provide additional context to each question: bi-directional communication, context, translation and tool/technology development.

### Bidirectional relationship of immune and nervous systems interactions

Interactions between the immune and nervous systems are bidirectional and both the neuroimmune and immune-neuro perspectives should be considered when addressing the research questions.

For example, in the immune-to-neuro direction, the presence of immune cells in the CNS was once considered a sign of neuropathology, but it is now increasingly recognised that immune signalling in the CNS is important for normal development and healthy brain function. The formation of mature neural circuits for example requires pruning of synapses by the immune system, including the classical complement pathway (initiated by C1q) ^9^, microglia ^10^ and MHC Class I ^11^. By extension, inappropriate activation of the immune system can lead to excessive synapse loss and neurodegenerative disease, including Alzheimer’s Disease ^12^. This fine balance of immune function in the CNS is further demonstrated by the ability of antigen-specific T cells to improve neuronal survival after a CNS injury ^13^.

In pathological states, such as immune-mediated inflammatory diseases and autoantibody-mediated neurological conditions, immune function is unequivocally involved in neuroinflammatory damage and/or pain but, paradoxically, can also support tissue regeneration (e.g. remyelination). Inflammation can also play a role in other states and conditions, such as stress resilience and post-traumatic stress disorder (PTSD). Immune-deficient mice (severe combined immunodeficiency and nude mice) were more likely to develop PTSD than wildtype mice when subjected to stress, with improvements seen in the stress response upon transfer of T cells from wildtype donors ^13^. Further roles are also proposed for T cells in learning memory and behaviour, in both antigen-specific and antigen-independent manners ^13^.

When considering areas to prioritise, it was noted that strategies modulating the immune system to improve neurological or behavioural function are more developed than vice versa. As such, these are perhaps more likely to be taken forward in the medium term for a variety of reasons, e.g. due to challenges in developing brain penetrant drugs. However, we fully acknowledge the importance of psychological, behavioural and physical interventions that act via the nervous system to modulate immune function and their potential to be harnessed for therapeutic benefit. It has been recognised that the modulation of neural function plays a role in regulating immune responses, for example as seen in the gut-brain axis as well as strategies to modulate neurotransmitters or neuropeptides to influence health ^14, 15^. The immune system is also susceptible to behavioural conditioning whereby pairing of a novel aversive taste stimulus (conditioned stimulus) with an immunosuppressive drug e.g. cyclosporin (unconditioned stimulus) results in the taste stimulus itself exerting immunosuppressive properties ^16, 17^. Proof-of-principle data suggest that a similar behavioural conditioning approach may support an immunosuppressive drug dose-reduction strategy in renal transplant patients ^18^.

Activation of both the autonomic nervous system and the hypothalamo-pituitary-adrenal axis have been demonstrated to affect the immune system both directly and indirectly. For example, lymphocytes express surface receptors for neurohormones and transmitters and are exposed to neurochemicals in lymphoid organs including the spleen and in peripheral blood. Indeed, directly activating dopaminergic neurons in the mouse ventral tegmental area and characterizing the subsequent immune response after exposure to *Escherichia coli* has shown an increase in both innate and adaptive immune responses ^19^. This was indexed by enhanced antibacterial activity of monocytes and macrophages, reduced bacterial load and a heightened T cell response in a mouse model of delayed-type hypersensitivity ^19^.

Thus, studying both neuroimmune and immune-neuro interactions will be critical to providing a holistic mechanistic understanding of these pathways that will ultimately form the foundations for innovative interventions. Furthermore, these established CNS-immune communication pathways demonstrate the potential of psychological/psycho-social interventions to improve immune health and the importance of thinking more broadly, i.e. beyond pharmacological modulators, about how neuroimmunology could inform strategies to support health.

### The importance of context

Understanding the context of neuroimmune cross-talk is critical when considering the underlying mechanisms. When tackling these priorities, research teams should carefully consider and report the rationale behind the chosen experimental context(s). For example, localisation (e.g. a specific brain region or peripheral nerve terminal), age or developmental stage, and/or health or disease setting, including relapse and remission. The context itself could define which research questions are a priority to address first, or which cell types to investigate and how. For example, studying a particular cell type might be most appropriate within a specific disease setting or developmental stage.

Investigating neuroimmune relationships during homeostasis and development from pregnancy and early life through to ageing will provide significant mechanistic insights into the interactions. While the intent of the discussion was to be broadly disease-agnostic, defining where and how disease is included in the investigations was recognised to be important. The disease context has the potential to provide fundamental insights for certain common phenotypes across multiple diseases, both with relevance to aetiology of disease onset, persistence and progression. For example, insight into brain development can give significant clues into mechanisms that can be reactivated in disease (for example synaptic elimination). Indeed, neuroinflammation is beneficial in the right context and so improving our understanding of when it switches from being beneficial (e.g. instructing developmental processes, removing debris, fighting infection, promoting regeneration) to detrimental (e.g. potential maladaptive synaptic pruning, failure to sense danger, uncontrolled inflammation) to neurological health, is required. Failure to consider different contexts could lead to the unintended exclusion of important areas and overlooking of key mechanisms, for instance natural changes in neuroimmune cross-talk during critical periods of development and ageing.

Contextual elements that should be considered include the effect of genetic background, risk factors and co-morbidities (e.g. metabolic disorders and obesity, or chronic low-grade infection and changes in the microbiome), all of which can lead to chronic inflammation and an impact on the nervous system, and predate disorders such as psychosis or depression ^20^. The influence of sleep and changes in neuroendocrine signalling (including glucocorticoids, androgens, oestrogens, neuropeptides and other hormones) and the impact of therapeutic interventions for chronic conditions (such as chemotherapy, immunomodulation or analgesics) are also important considerations. The impact of diversity on neuroimmune interactions, including sex and ethnicity, will also be important in gaining real understanding of the nuances of these interactions. Secondary influences, such as environmental factors, pollution, exercise, epidemics, therapeutics, poverty or stress, are increasingly recognised as playing important roles in shaping these interactions. As well as encouraging new epidemiological studies, the impact of societal factors opens the way for new collaborations with experts in the social sciences, further breaking down traditional academic siloes. While the impact of acute and chronic infection was not addressed directly when developing the priorities, infection and neuroimmune interactions are inherently linked e.g. in the maternal-immune activation model, where prenatal exposure to infection could be a driver in initiating depression or psychosis in later life, or chronic gum disease as a driver of dementia ^21^.

In summary, whilst reductionist and mechanistic experimental studies are pivotal, the impact of neuroimmune interactions cannot be studied in isolation, and the broader context of these interactions, be it co-morbidities, age, chronic stress or infection, need to be taken into consideration (and reported) when trying to understand the roles and functions of these interactions over time. This can add a level of complexity but is critical in providing a complete understanding.

### Tools/technology development

Addressing some of the priority questions fully will require development of new tools and technologies. While there have been major advances in recent years, there will be an increasing need to continue to develop sensitive and selective tools to measure and modulate immune cells and molecules *in vivo*, particularly within the CNS. This applies to both human and animal models to study interactions in homeostasis and development as well as in disorders, where a lack of suitable tools often presents a major barrier to progress. For instance, being able to image and modulate CNS-resident or CNS-infiltrating immune cells and pathways in the living nervous system, without affecting the peripheral immune system, would be game changing, allowing questions to be asked that are not currently addressable around the dynamics of these cells and pathways *in vivo*. Genetic tools, robust target-specific monoclonal antibodies, novel biological labels and synthetic biology may all contribute to the new toolbox.

Analysis of the full repertoire of immune cells and molecules resident in the brain and nervous system will greatly benefit from the generation of detailed cell atlases that incorporate study of the peripheral immune system. This, however, may require development of new or more specific markers to study the different immune cell types in the first instance, and then progress to specific tools to track and manipulate cellular behaviour. The migratory nature and dynamic aspects of cell phenotypes of the immune compartment may provide additional challenges to cell atlas development.

Collaborations beyond the biological sciences could be one way forward to develop or optimise these much-needed methods and tools. For example, working with medical physicists to develop neuroimaging tools sensitive to discrete CNS immune cell types or with bioengineers to develop cell type-specific targeting vehicles that could deliver pharmacological modulators directly to cells of interest, would be transformative from both a discovery and clinical perspective. Computational approaches are equally needed in order to integrate and analyse the large amount of clinical and basic research data generated and develop hypotheses for further experimental testing. This includes neuroimaging and biomarker data, eHealth records and the outputs from large scale ‘omics approaches.

### Translation to the clinic

Dissecting fundamental questions of neuroimmune interactions, such as those proposed here, can lead to an improved understanding of both systems and how disordered interactions can be potentially causal in major neurological disorders and mental illnesses. Increasing translational potential requires investigating changes in neuronal circuitry, synaptic plasticity, CNS development and ageing, and homeostasis and (dys)function in both human and model systems. Studying effects of immune-modulating therapies on the nervous system, behaviour, and psychopathology can help to elucidate pathophysiologic mechanisms, leading to development of novel or repurposed immunotherapies. Progress in this area has been greatest in multiple sclerosis. Several immune-modulating drugs are now available to effectively delay progression of neurodegeneration and work by influencing peripheral immune cell trafficking to the CNS or modulating immune cell activation. Natalizumab (anti-alpha-4 integrin) has been shown to block entry of peripheral immune cells into the CNS, alleviating disease progression and further highlighting the importance of studying interactions between the peripheral immune system and the CNS for therapeutic gain ^22^. The high level of specificity conferred by the autoantibody-mediated diseases of the nervous system offer a direct link between neuroscience and immunology, allowing their parallel study in humans with these diseases ^23^.

In psychiatry, interleukin (IL)-6 has been identified as a potential target in patients with depression and schizophrenia using population cohort and genetic studies ^24, 25^. However, patients receiving IL-6 receptor blockade (tocilizumab) as acute graft-versus-host-disease prophylaxis experienced significantly more depressive symptoms ^26^. This indicates that further investigation is required to test the therapeutic potential of targeting the immune system specifically in patients with depression, such as the Insight study, a proof-of-concept experimental medicine trial of tocilizumab ^27^. Emerging evidence has also demonstrated that increased inflammation may identify the phenotypic profile of patients more likely to benefit from anti-inflammatory augmentation across depression and psychosis ^28, 29^. This highlights how, when testing an immune-modulatory drug in a complex disease like depression (or dementia), it is essential to first demonstrate that inflammation is occurring and stratify inflammatory patients to the therapy. Successful translation to the clinic will require evidence triangulation using different approaches. Inflammation is likely to be relevant for some, but not all patients with mental health disorders, and not at all stages of illness. Further work is needed to understand the characteristics of inflammation-related depression and psychosis in order to inform patient selection in future interventional studies, thereby improving trial readouts, which is critical for clinical translation.

Furthermore, developing systems to experimentally suppress or stimulate the immune system under controlled conditions can provide a platform to study neuroimmune dysregulation or understand its function in the CNS. However, this will require standardisation of clinical parameters to aid comparative studies and interoperability of cohort data, for example standardisation of methods for measurement of neurological features (fatigue, pain, cognition) and protocols for collecting and processing samples for immune characterisation. Achieving this goal would be facilitated by closer collaboration between research teams.

## Conclusion

Neuroimmunology is an expansive field, which covers a vast breadth of science from fundamental interactions of cells to the effects of behaviour on the immune system. Bringing together different research communities is therefore critical to making progress, ensuring a joined-up approach with sharing of knowledge and learning. With these proposed research priorities for the field, we hope to provide a focal point for teams, galvanise collective endeavours and move neuroimmunology forward as a whole. The importance of considering the impact of context when addressing these questions is also highlighted. With multiple groups tackling each problem from different, but complementary or even synergistic angles, together they will provide an ever more granular picture of how interactions between the immune and nervous systems influence health and disease. By defining the underpinning and causal mechanisms through basic science as well as translational research in human cohorts, we anticipate impacting on health and facilitating the discovery of new diagnostic and therapeutic targets.

Achieving this vision will require the continued development of new and improved tools, open sharing and curation of data sets, multi- and inter-disciplinary teams working with colleagues in the wider biological sciences, STEM and social sciences, as well as forging partnerships with clinicians, patients and industry.

Finally, these research priorities were developed by the research community, for the research community, as an attempt to identify the areas that most urgently need addressing to ultimately improve health, to start conversations and new collaborations, and coordinate existing efforts. By building upon the current strong knowledge base and bringing in new disciplines and perspectives, we hope to inspire not only new lines of enquiry but also encourage researchers from diverse backgrounds to become neuroimmunologists. Together, we can further our understanding of this increasingly important field which has the potential to make a major impact on health.

## Data availability

No data are associated with this article.

## References

[1] Bohlen CJ Friedman BA Dejanovic B : Microglia in Brain Development, Homeostasis, and Neurodegeneration. *Annu Rev Genet.* 2019;53:263–288. 10.1146/annurev-genet-112618-043515 31518519

[2] Kalambogias J Chen CC Khan S : Development and sensory experience dependent regulation of microglia in barrel cortex. *J Comp Neurol.* 2020;528(4):559–573. 10.1002/cne.24771 31502243 PMC6944757

[3] Szepesi Z Manouchehrian O Bachiller S : Bidirectional Microglia-Neuron Communication in Health and Disease. *Front Cell Neurosci.* 2018;12:323. 10.3389/fncel.2018.00323 30319362 PMC6170615

[4] John CC Carabin H Montano SM : Global research priorities for infections that affect the nervous system. *Nature.* 2015;527(7578):S178–86. 10.1038/nature16033 26580325 PMC4697933

[5] Taquet M Luciano S Geddes JR : Bidirectional associations between COVID-19 and psychiatric disorder: retrospective cohort studies of 62 354 COVID-19 cases in the USA. *Lancet Psychiatry.* 2021;8(2):130–140. 10.1016/S2215-0366(20)30462-4 33181098 PMC7820108

[6] The Lancet Neurology: Long COVID: understanding the neurological effects. *Lancet Neurol.* 2021;20(4):247. 10.1016/S1474-4422(21)00059-4 33743226 PMC7969137

[7] Iadecola C Anrather J Kamel H : Effects of COVID-19 on the Nervous System. *Cell.* 2020;183(1):16–27.e1. 10.1016/j.cell.2020.08.028 32882182 PMC7437501

[8] Caldwell LJ Subramaniam S MacKenzie G : Maximising the potential of neuroimmunology. *Brain Behav Immun.* 2020;87:189–192. 10.1016/j.bbi.2020.03.010 32201255 PMC8353661

[9] Stevens B Allen NJ Vazquez LE : The classical complement cascade mediates CNS synapse elimination. *Cell.* 2007;131(6):1164–78. 10.1016/j.cell.2007.10.036 18083105

[10] Schafer DP Lehrman EK Kautzman AG : Microglia sculpt postnatal neural circuits in an activity and complement-dependent manner. *Neuron.* 2012;74(4):691–705. 10.1016/j.neuron.2012.03.026 22632727 PMC3528177

[11] Huh GS Boulanger LM Du H : Functional requirement for class I MHC in CNS development and plasticity. *Science.* 2000;290(5499):2155–9. 10.1126/science.290.5499.2155 11118151 PMC2175035

[12] Hong S Beja-Glasser VF Nfonoyim BM : Complement and microglia mediate early synapse loss in Alzheimer mouse models. *Science.* 2016;352(6286):712–716. 10.1126/science.aad8373 27033548 PMC5094372

[13] Kipnis J Gadani S Derecki NC : Pro-cognitive properties of T cells. *Nat Rev Immunol.* 2012;12(9):663–9. 10.1038/nri3280 22903149 PMC4032225

[14] Brinkman DJ Ten Hove AS Vervoordeldonk MJ : Neuroimmune Interactions in the Gut and Their Significance for Intestinal Immunity. *Cells.* 2019;8(7):670. 10.3390/cells8070670 31269754 PMC6679154

[15] Koopman FA Chavan SS Miljko S : Vagus nerve stimulation inhibits cytokine production and attenuates disease severity in rheumatoid arthritis. *Proc Natl Acad Sci U S A.* 2016;113(29):8284–9. 10.1073/pnas.1605635113 27382171 PMC4961187

[16] Hadamitzky M Lückemann L Pacheco-López G : Pavlovian Conditioning of Immunological and Neuroendocrine Functions. *Physiol Rev.* 2020;100(1):357–405. 10.1152/physrev.00033.2018 31437089

[17] Ader R Cohen N : Behaviorally conditioned immunosuppression. *Psychosom Med.* 1975;37(4):333–40. 10.1097/00006842-197507000-00007 1162023

[18] Kirchhof J Petrakova L Brinkhoff A : Learned immunosuppressive placebo responses in renal transplant patients. *Proc Natl Acad Sci U S A.* 2018;115(16):4223–4227. 10.1073/pnas.1720548115 29610294 PMC5910853

[19] Ben-Shaanan TL Azulay-Debby H Dubovik T : Activation of the reward system boosts innate and adaptive immunity. *Nat Med.* 2016;22(8):940–4. 10.1038/nm.4133 27376577

[20] Perry BI Stochl J Upthegrove R : Longitudinal Trends in Childhood Insulin Levels and Body Mass Index and Associations With Risks of Psychosis and Depression in Young Adults. *JAMA Psychiatry.* 2021;78(4):416–425. 10.1001/jamapsychiatry.2020.4180 33439216 PMC7807390

[21] Dominy SS Lynch C Ermini F : *Porphyromonas gingivalis* in Alzheimer's disease brains: Evidence for disease causation and treatment with small-molecule inhibitors. *Sci Adv.* 2019;5(1):eaau3333. 10.1126/sciadv.aau3333 30746447 PMC6357742

[22] Brandstadter R Katz Sand I : The use of natalizumab for multiple sclerosis. *Neuropsychiatr Dis Treat.* 2017;13:1691–1702. 10.2147/NDT.S114636 28721050 PMC5499927

[23] Sun B Ramberger M O'Connor KC : The B cell immunobiology that underlies CNS autoantibody-mediated diseases. *Nat Rev Neurol.* 2020;16(9):481–492. 10.1038/s41582-020-0381-z 32724223 PMC9364389

[24] Khandaker GM Pearson RM Zammit S : Association of serum interleukin 6 and C-reactive protein in childhood with depression and psychosis in young adult life: a population-based longitudinal study. *JAMA Psychiatry.* 2014;71(10):1121–8. 10.1001/jamapsychiatry.2014.1332 25133871 PMC4561502

[25] Khandaker GM Zuber V Rees JMB : Shared mechanisms between coronary heart disease and depression: findings from a large UK general population-based cohort. *Mol Psychiatry.* 2020;25(7):1477–1486. 10.1038/s41380-019-0395-3 30886334 PMC7303009

[26] Knight JM Costanzo ES Singh S : The IL-6 antagonist tocilizumab is associated with worse depression and related symptoms in the medically ill. *Transl Psychiatry.* 2021;11(1):58. 10.1038/s41398-020-01164-y 33462203 PMC7812704

[27] Khandaker GM Oltean BP Kaser M : Protocol for the insight study: a randomised controlled trial of single-dose tocilizumab in patients with depression and low-grade inflammation. *BMJ Open.* 2018;8(9):e025333. 10.1136/bmjopen-2018-025333 30244217 PMC6157523

[28] Nettis MA Lombardo G Hastings C : Augmentation therapy with minocycline in treatment-resistant depression patients with low-grade peripheral inflammation: results from a double-blind randomised clinical trial. *Neuropsychopharmacology.* 2021;46(5):939–948. 10.1038/s41386-020-00948-6 33504955 PMC8096832

[29] Krynicki CR Dazzan P Pariante CM : Deconstructing depression and negative symptoms of schizophrenia; differential and longitudinal immune correlates, and response to minocycline treatment. *Brain Behav Immun.* 2021;91:498–504. 10.1016/j.bbi.2020.10.026 33161162

