# Supplementary material for: Research priorities for neuroimmunology: identifying the key research questions to be addressed by 2030
Source: Wellcome Open Res. 2021 Jul 29;6:194. [Version 1] doi: 10.12688/wellcomeopenres.16997.1 (PMC8558843; doi:10.12688/wellcomeopenres.16997.1)
Supplement: Peer review history

# Peer review: Reviewer response for version 1

**DOI:** 10.21956/wellcomeopenres.18768.r46450

**Published:** 2021-10-29

**Authors:** Keith Kelley

**Affiliations:** 1 Department of Pathology, College of Medicine, University of Illinois at Urbana-Champaign, Urbana, IL, USA

There is a lack of visibility of neuroimmunology in both the medical literature and its translation into clinical practice. Seventeen very well-respected scientists from the United Kingdom came together to address these and other issues. They lay the groundwork for a path forward to move biomedical research on communication systems between the central nervous and immune systems into the clinic. The authors do a good job of acknowledging and documenting the progress in basic research made in this field during the past few decades. Nevertheless, there is a real need to further support this rapidly expanding field by trying to understand reciprocal systems of communication that are now known to exist between the immune system and brain. The authors make a compelling recommendation to create teams from a variety of academic disciplines to accomplish this goal and to advance the field. I agree and support all of this.

 It is admirable for a group of esteemed scholars to come together, presumably virtually in October 2020 during the COVID19 pandemic. As a result, they agreed to include their names on a paper that boldly proposes ten approaches, all to be accomplished within 10 years, to achieve their goals. The authors propose ten questions within four different frameworks: (a) The context of understanding the importance of bidirectional communication (e.g., afferent vs efferent); (b) Deciphering whether neuroimmune crosstalk is more important in some contexts that others (e.g., development vs aging) and the contribution of co-morbidities; (c) The need for advancement and development of newer technologies to non-invasively assess neuroimmune interactions in animals and humans; and (d) The importance of translating answers to these ten fundamental questions into treatments that help patients. These are all reasonable and important issues for discussion in health and disease. If accomplished, the entire neuroimmunology field will be much stronger in 2031 than it is now 2021.

 This is a well-written and persuasive article, but it could be strengthened. Although top-down (efferent) pathways are mentioned, the clear focus of this article is on bottom-up communication from the immune system to the brain, with the aim of improving/understanding/treating mental health disorders. There is only one question that directly asks how neural circuits, presumably from both the central and peripheral nervous systems, affect immunity. Yet, the recent review by Aysa Rolls and colleagues makes a convincing argument for brain regulation of a variety of immune events ( https://doi.org/10.1038/s41577-020-0387-1). ^1^ As such, the case for efferent regulation of the immune system by the brain could be stronger.

 Natural ways to exploit immune-brain communication to promote health, as opposed to treatment with drugs, could be discussed in more detail. For example, Western medicine finally has recognized that there are non-pharmaceutical approaches that can improve mental health disorders. They are mindfulness and exercise in all forms, with the practice of mindfulness now blending with traditional Chinese meditation and exercise. Other aspects of Eastern medicine also offer promise. For example, a major criticism of the use of acupuncture for the treatment of human diseases is the lack of an anatomical pathway to mediate the effects of specific acupoints. That critique will now change with the recent publication in *Nature* that defined a neuroanatomical loop between the periphery and brain and the subsequent efferent signal that defines the manner by which a specific acupoint inhibits LPS-induced inflammation ( https://doi.org/10.1038/s41586-021-04001-4). ^2^

 Finally, it is curious that the noun “physiology” is never mentioned throughout the entire paper. Yet systemic physiology is based not only on structure and function but also on communication systems among various organs in the body. Lungs communicate with the brain to regulate blood gas levels and the heart communicates with the lungs for the same reason. The kidney and lungs communicate with one another to maintain acid-base balance. These systems form regulatory feedback loops, which control everything from reproductive cycles to stress responses. It seems that, in principle, immune-brain communication would not be much different from these well-accepted autonomic reflexes. Yet this paper seems to present immune-brain communication as a one-way street, which is either afferent or efferent. As such, more emphasis could be given to regulatory feedback loops. For example, over thirty years ago Hugo Besedovsky discovered one of these feedback systems by showing that a peripheral inflammatory stimulus (IL-1) caused release of corticotropin-releasing factor from the hypothalamus, ultimately leading to the release of corticosterone from the adrenal cortex ( *Science* 1987; 238:524-526). These data defined a clear immune afferent signal to the brain that caused an efferent endocrine response that in turn regulates some aspect of the immune system. One can always quibble about the ten questions that the authors propose to lead the field forward. But perhaps the authors might consider asking an eleventh question: What are the important physiological immune-brain feedback regulatory control loops and how are they affected throughout the life span and in health and disease?

Does the article adequately reference differing views and opinions?

Yes

Are all factual statements correct, and are statements and arguments made adequately supported by citations?

Yes

Is the Open Letter written in accessible language?

Yes

Where applicable, are recommendations and next steps explained clearly for others to follow?

Yes

Is the rationale for the Open Letter provided in sufficient detail?

Yes

Reviewer Expertise:

Immunophysiology

I confirm that I have read this submission and believe that I have an appropriate level of expertise to confirm that it is of an acceptable scientific standard.

## References

[1] : Neuronal regulation of immunity: why, how and where?. *Nat Rev Immunol* .21(1) : 10.1038/s41577-020-0387-1 20-36 10.1038/s41577-020-0387-1 32811994

[2] : A neuroanatomical basis for electroacupuncture to drive the vagal-adrenal axis. *Nature* .2021;598(7882) : 10.1038/s41586-021-04001-4 641-645 10.1038/s41586-021-04001-4 34646018PMC9178665

# Peer review: Reviewer response for version 1

**DOI:** 10.21956/wellcomeopenres.18768.r45229

**Published:** 2021-09-06

**Authors:** Asya Rolls

**Affiliations:** 1 Department of Immunology, Rappaport Faculty of Medicine, Technion-Israel Institute of Technology, Haifa, Israel

In this manuscript, the authors provide an overview of research directions in neuroimmunology. It's a very clear manuscript, highlighting the key research directions and main challenges in addressing them. The authors explain the rationale for selecting the directions they propose and discuss the main gaps in the field. It's a very timely piece and it summarizes the views of the authors. Thus, I can only offer a few suggestions, based on my experience as a reader, on how to increase its accessibility. 

1. In the section "importance of context" -adding references to articles that exemplify the proposed research directions, can help the readers better appreciate the potential of the field. 
2. For the technology part, an illustration of the challenges can be helpful. 
3. Adding more questions on how the nervous system affects immunity to Figure 1 will emphasize the bi-directional nature of the interactions.  For example, how do different forms of brain activity (different areas, states) affect immune activity? Which neuronal mediators can be secreted to the periphery? 

Does the article adequately reference differing views and opinions?

Yes

Are all factual statements correct, and are statements and arguments made adequately supported by citations?

Yes

Is the Open Letter written in accessible language?

Yes

Where applicable, are recommendations and next steps explained clearly for others to follow?

Yes

Is the rationale for the Open Letter provided in sufficient detail?

Yes

Reviewer Expertise:

Neuoimmunology

I confirm that I have read this submission and believe that I have an appropriate level of expertise to confirm that it is of an acceptable scientific standard.
